# Chlamydial Induction of Hydrosalpinx in 11 Strains of Mice Reveals Multiple Host Mechanisms for Preventing Upper Genital Tract Pathology

**DOI:** 10.1371/journal.pone.0095076

**Published:** 2014-04-15

**Authors:** Jianlin Chen, Hongbo Zhang, Zhou Zhou, Zhangsheng Yang, Yiling Ding, Zhiguang Zhou, Edward Zhong, Bernard Arulanandam, Joel Baseman, Guangming Zhong

**Affiliations:** 1 Department of Microbiology and Immunology, University of Texas Health Science Center at San Antonio, San Antonio, Texas, United States of America; 2 Departments of Obstetrics and Gynecology, Pathology and Endocrinology, Second Xiangya Hospital, Central South University, Changsha, Hunan, China; 3 Department of Economics, University of Wisconsin-Madison, Madison, Wisconsin, United States of America; 4 Department of Biology, University of Texas at San Antonio, San Antonio, Texas, United States of America; University of Louisville, United States of America

## Abstract

The female lower genital tract is constantly exposed to microbial infection, some of which can ascend to and cause pathology such as hydrosalpinx in the upper genital tract, which can affect fertility. To understand host mechanisms for preventing upper genital tract pathology, we screened 11 inbred strains of mice for hydrosalpinx induction by *C. muridarum*. When examined on days 60 to 80 after intravaginal infection, the 11 strains fell into 3 groups based on their hydrosalpinx severity: CBA/J and SJL/J mice were highly susceptible with a hydrosalpinx score of 5 or greater; Balb/c, C57BL/6J, C57BL/10J, C3H/HeJ and C3H/HeN were susceptible with a score between 1 and <5; NOD/ShiLtJ, DBA/1J, DBA/2J and A/J were resistant with a score of <1. The diverse range of mouse susceptibility to hydrosalpinx induction may reflect the varied clinical outcomes of *C. trachomatis*-infected women. When the 11 strains were infected via an intrauterine inoculation to bypass the requirement for ascension, higher incidence and more severe hydrosalpinges were induced in most mice, indicating that the interaction between chlamydial ascension and host control of ascension is critical for determining susceptibility to hydrosalpinx development in many mice. However, a few mouse strains resisted significant exacerbation of hydrosalpinx by intrauterine infection, indicating that these mice have evolved ascension-independent mechanisms for preventing upper genital tract pathology. Together, the above observations have demonstrated that different strains of mice can prevent upper genital tract pathology by using different mechanisms.

## Introduction

Sexually transmitted infection with *Chlamydia trachomatis* in the lower genital tract can lead to upper genital tract pathology such as hydrosalpinx, resulting in clinical complications including infertility in some women [1]. To preserve fertility, women must have evolved mechanisms for protecting the upper genital tract from infection-associated pathology. Although both *C. trachomatis* ascension and *C. trachomatis* infection-induced inflammatory responses are believed to contribute significantly to the upper genital pathology [2], [3], the relative roles of ascension and inflammation in different women with upper genital tract pathology are unknown and the biological mechanisms on how most women infected with *C. trachomatis* do not develop upper genital tract pathology remain unclear. *Chlamydia muridarum* organisms have been extensively used to study the mechanisms of *C. trachomatis* pathogenesis [4]. This is because genital tract infection of mice with *C. muridarum* can cause hydrosalpinx, which closely mimics the tubal pathology induced by *C. trachomatis* in humans. Hydrosalpinx has been considered a surrogate marker for tubal occlusion and tubal factor infertility [5]–[7]. Thus, investigating how female mice respond to *C. muridarum* infection by monitoring hydrosalpinx development may allow us to learn the mechanisms used by Chlamydia and host for inducing and preventing upper genital tract pathology, respectively.

Using *C. muridarum* intravaginal infection mouse model, various host innate and adaptive immunity components have been identified to play significant roles in upper genital tract pathology [8]–[10]. However, the roles of the interactions between chlamydial ascension and mouse control of ascending infection have not been addressed. Furthermore, Chlamydia-triggered hydrosalpinx-causing inflammation and mouse regulation of the inflammatory responses in hydrosalpinx development need clarification.

The fact that only a portion of women infected with *C. trachomatis* develops the upper genital pathology hydrosalpinx has led to the comparison of host immune responses between *C. trachomatis*-infected women with or without tubal factor infertility [11], [12]. These clinical studies have revealed the association of chlamydial antigen-specific antibody responses with tubal factor infertility, which may help to develop biomarkers for diagnosing or predicting Chlamydia-induced infertility. However, it has been difficult to investigate the interactions between chlamydial ascension and host control of ascension in humans. Variations in hydrosalpinx development upon *C. muridarum* infection between different strains of mice have been noted before [3], [5], [13], [14]. However, the varied susceptibility to hydrosalpinx induction by chlamydial infection has not been systematically utilized for studying the roles of host and chlamydial mechanisms in the development of upper genital tract pathology.

In the current study, we compared 11 inbred strains of mice for their susceptibility to hydrosalpinx induction by *C. muridarum* infection in the lower versus upper genital tracts. Lower genital infection via an intravaginal inoculation (mimicking sexually transmitted infection in humans) has revealed a wide range of mouse susceptibility to hydrosalpinx induction with some strains highly susceptible while others resistant. The full range of susceptibility to hydrosalpinx induction among the 11 strains of mice may reflect the diverse outcomes in women urogenitally infected with *C. trachomatis*. To investigate the contribution of ascending infection and the role of host control of ascending infection in hydrosalpinx development, we took advantage of an intrauterine inoculation for delivering the same amounts of *C. muridarum* organisms directly into the upper genital tract [13]. By circumventing the requirement for ascending infection, the intrauterine inoculation increased hydrosalpinx incidence and enhanced hydrosalpinx severity in most mice, suggesting that the interactions between chlamydial ascension and host control of chlamydial ascension play significant roles in determining hydrosalpinx susceptibility. However, several strains of mice resisted the enhancement of hydrosalpinx induction by the intrauterine route of infection, suggesting that ascension-independent mechanisms are more important for regulating hydrosalpinx development in these mice. Thus, it is likely that different mouse strains (and individual women) may use different mechanisms for preventing hydrosalpinx.

## Materials and Methods

### 1. Chlamydial organisms and infection

The *C. muridarum* organisms (Nigg strain) used in the current study were propagated in HeLa cells (human cervical carcinoma epithelial cells, ATCC cat# CCL2.1), purified, aliquoted and stored as described previously [3], [15]. Female CBA/J (Jackson Laboratories; stock number 000656), SJL/J (000686), Balb/cJ (000651), C57BL/6J (000664), C3H/HeJ (000659), C57BL/10J (000665), NOD/ShiLtJ (001976), DBA/1J (000670), DBA/2J (000671), A/J (000646) and C3H/HeN (Charles River) were purchased at the age of 5 to 6 weeks old from Jackson Laboratories (Bar Harbor, Maine) or Charles River Laboratories, Wilmington, MA. Each mouse was inoculated intravaginally with 2×10^5^ IFUs of live *C. muridarum* organisms in 20 µl of SPG (sucrose-phosphate-glutamate buffer) or via intrauterine with the same amount of organisms but in 3 µl of SPG. The animal experiments were carried out in accordance with the recommendations in the Guide for the Care and Use of Laboratory Animals of the National Institutes of Health. The protocol was approved by the Committee on the Ethics of Laboratory Animal Experiments of the University of Texas Health Science Center at San Antonio.

For intravaginal inoculation, the inoculum was delivered into mouse vagina using a 200 µl micropipette tip as described previously [3]. Carefully and gently insert the tip into mouse vagina until slight resistance was felt. Then, press the pipette plunger completely to release all inoculum followed by gentle withdrawing of the tip without releasing the pipette plunger.

For intrauterine or transcervical inoculation, a Non-Surgical Embryo Transfer Device (NSET, cat# 60010, ParaTechs Corp., Lexington, KY) was used and the manufacturer's instruction (http://www.paratechs.com/nset/) was followed. Briefly, after connecting the NSET device onto a GeneMate P10 micropipette (0.5–10 µl size, BioExpress, Kaysville, UT), the micropipette was used to take up 3 µl inoculum solution and then carefully adjusted to a setting of 3.5 µl to generate a small air bubble at the tip of the NSET. A speculum was gently placed into the mouse vagina to open up the vagina. The NSET loaded with the 3 µl inoculum was inserted into the speculum and through the cervix. The inoculum was delivered by pressing the pipette plunger completely. The NSET device was immediately and gently removed without releasing the pipette plunger, followed by removal of the speculum.

Although it was not possible to know which side of oviducts was infected by the inoculated organisms via either intravaginal or intrauterine routes, we always made sure that consistency in operating procedures was maintained by allowing one individual to infect all mice in a given experiment. For both routes, five days prior to the inoculation either intravaginally or via intrauterine injection, each mouse was injected subcutaneously with 2.5 mg Depo-Provera (Pharmacia Upjohn, Kalamazoo, MI) to synchronize estrus cycle and increase mouse susceptibility to chlamydial infection.

For *in vitro* infection of HeLa cells, HeLa cells grown on coverslips in 24-well plates containing DMEM (GIBCO BRL, Rockville, MD) with 10% fetal calf serum (FCS; GIBCO BRL) at 37°C in an incubator supplied with 5% CO_2_ were inoculated with *C. muridarum* organisms as described previously [3], [15]. The infected cultures were processed for immunofluorescence assay as described below.

### 2. Monitoring live *C. muridarum* organism recovery from swabs

To monitor live organism shedding, vaginal swabs were taken on different days after the intravaginal or intracervical infection. Each swab was suspended in 500 µl of ice-cold SPG followed by vortexing with glass beads, and the released organisms were titrated on HeLa cell monolayers in duplicates as described previously [3]. The total number of IFUs per swab/tissue was calculated based on the number of IFUs per field, number of fields per coverslip, dilution factors and inoculation and total sample volumes. An average was taken from the serially diluted and duplicate samples for any given swab/tissue. The calculated total number of IFUs/swab or tissue was converted into log_10_ and the log_10_ IFUs were used to calculate means and standard deviation for each group at each time point.

### 3. Evaluating mouse genital tract tissue pathologies and histological scoring

Mice were sacrificed on different days post infection as indicated in individual experiments, and the mouse urogenital tract tissues were isolated. Before the tissues were removed from the mouse body, an *in situ* gross examination was performed for evidence of oviduct hydrosalpinx or any other related abnormalities of oviducts. The severity of oviduct hydrosalpinx was scored based on the following criteria: 0, no hydrosalpinx; 1, hydrosalpinx detectable visually but requiring microscopic confirmation; 2, hydrosalpinx clearly visible with naked eyes but the size is smaller than the ovary on the same side; 3, equal to the ovary on the same side; 4, hydrosalpinx larger than the ovary on the same side. After photographing the excised tissues were fixed in 10% neutral formalin, embedded in paraffin and serially sectioned longitudinally (with 5 µm/each section). Efforts were made to include cervix, both uterine horns and oviducts as well as lumenal structures of each tissue in each section. The sections were stained with hematoxylin and eosin (H&E) as described elsewhere [5]. The H&E stained sections were assessed by a pathologist blinded to mouse treatment and scored for severity of inflammation and pathologies based on the modified schemes established previously [5], [14]. The uterine horns and oviducts were scored separately (only the oviduct scores were used in the current study). Scoring for dilation of uterine horn or oviduct: 0, no significant dilatation; 1, mild dilation of a single cross section; 2, one to three dilated cross sections; 3, more than three dilated cross sections; and 4, confluent-pronounced dilation. Scoring for chronic inflammatory cell infiltrates (at the chronic stage of infection, the infiltrates mainly contain mononuclear cells while at the acute stage, neutrophils dominate the infiltration): 0, no significant infiltration; 1, infiltration at a single focus; 2, infiltration at two to four foci; 3, infiltration at more than four foci; and 4, confluent infiltration. Scores assigned to individual mice were calculated into means ± standard errors for each group of animals. It is worth noting that all pathology scoring was carried out in double blind manner.

### 4. Immunofluorescence assay

HeLa cells grown on coverslips with chlamydial infection were fixed and permeabilized for immunostaining as described previously [16], [17]. Hoechst dye (blue, Sigma) was used to visualize nuclear DNA. For titrating IFUs from swab and tissue homogenate samples, a mouse anti-chlamydial LPS antibody (clone# MB5H9, unpublished observation) plus a goat anti-mouse IgG conjugated with Cy3 (red; Jackson ImmunoResearch Laboratories, Inc., West Grove, PA) were used to visualize chlamydial inclusions. All immunofluorescence-labeled samples were observed under an Olympus AX-70 fluorescence microscope equipped with multiple filter sets (Olympus, Melville, NY).

### 5. Cytokine measurements in oviduct tissue homogenate samples

Oviduct/ovary tissues were harvested from highly susceptible strain CBA/J (n = 5) and resistant strain A/J (n = 5) on day 14 after intravaginal inoculation with *C. muridarum* for making homogenates as described previously [13], [14]. The homogenates were used for simultaneous measurements of 32 mouse cytokines [23 plex group I (cat# M60-009RDPD) plus 9 plex group II (MD0-00000EL)] using a multiplex bead array assay (Bio-Plex 200 System) all from Bio-Rad (Hercules, CA 94547) by following the manufacturer's instruction. All cytokines were expressed in pg/ml as mean plus/minus standard deviation. The means from the two mouse strains were used for calculating ratio and statistics analysis (Student *t*-Test). A ratio of CBA/J versus A/J above 4 is highlighted in bold face. Note that 27 of the 32 cytokines were significantly higher in oviducts of highly susceptible CBA/J than those of resistant A/J mice, 14 of which displayed more than 4 folds difference.

### 6. Statistical analyses

The pathology score data were analyzed with Wilcoxon rank sum test. The Fisher's Exact test was used to analyze category data including the % of mice with oviduct hydrosalpinx. The Student *t*-Test was used to compare cytokine concentrations between different groups. The Spearman's Correlation was used to analyze relationships between the different parameters describing mouse oviduct pathology and between oviduct pathology and lower genital tract infection following inoculation via either intrauterine or intravaginal routes. The lower genital tract infection was expressed as either recovered IFUs on different days after infection, days required for completely clearing infection or the filled areas under the corresponding live organism shedding curves. The filled area was calculated as the sum of all IFUs after each IFU times the corresponding number of days when the IFU was recovered.

## Results

### 1. Infection at the lower genital tract via intravaginal inoculation reveals distinct susceptibility to hydrosalpinx induction among 11 different strains of mice

**Figure 1:**
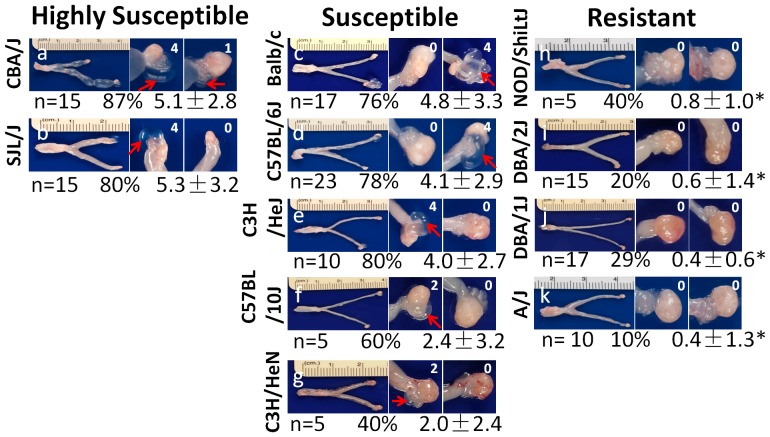
Hydrosalpinx development in 11 strains of mice following lower genital tract infection with *C. muridarum*. Eleven different strains of female mice as indicated on the left of each representative image (with n = 5 to 23 mice in each strain as marked below each image) were intravaginally infected with *C. muridarum*. Sixty to 80 days after infection, all mice were sacrificed for observing hydrosalpinx. A representative image from each strain of mice is presented with the whole genital tract in the left and the magnified oviduct/ovary portion in the right. The number of mice with positive hydrosalpinx (as marked with red arrows in the magnified oviduct/ovary images) were counted and recorded as % of hydrosalpinx-positive mice. Furthermore, the severity of each hydrosalpinx was scored based on the criteria described in the Materials and Methods and marked with numbers in white in the corresponding images. The hydrosalpinx severity scores were compared between different strains of mice using Wilcoxon Rank Sum while the hydrosalpinx incidences were compared using Fisher's Exact. Note that the hydrosalpinx scores of NOD/ShiLtJ (panel h), DBA/1J (i), DBA/2J (j) and A/J (k) mice were <1 and significantly lower than those from other strains of mice (as noted with stars, p<0.05). These four strains were designated “resistant” to hydrosalpinx induction (right column) while the CBA/J (a) and SJL/J (b) mice with the highest rates and the most severe hydrosalpinx was designated “highly susceptible” (left column). The remaining strains all developed significant hydrosalpinx, thus were designated as “susceptible” (c–g in the central column).

**Figure 2:**
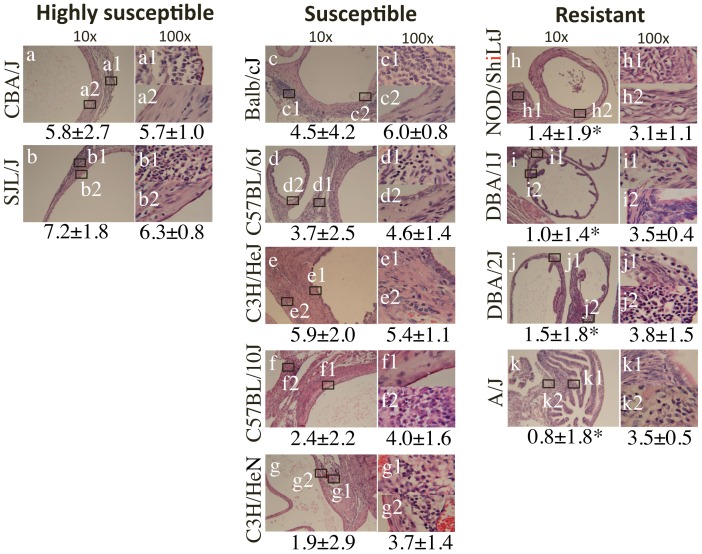
Hydrosalpinx examined under microscopy. The same genital tract tissues described in Fig. 1 legend were subjected to sectioning for H&E staining and microscopic observation. Both lumenal dilation and inflammatory infiltration were scored for oviduct tissues. Representative images from each group of mice taken under 10× (left columns, panels a–k) and 100× (right column, panels a1-k2) objective lens respectively are shown. The rectangles in each 10× image indicate where the two 100× images shown on the right were taken. Both the oviduct lumenal dilation (left) and inflammatory infiltration (right) scores (mean plus/minus standard deviation) from each group are listed under the corresponding images. Note that the lumenal dilation scores were significantly lower in the resistant strains (panels h–k) than highly susceptible (a & b) and 3 of 5 susceptible strains of mice (c–e). *p<0.05

Sixty to 80 days after the 11 different strains of mice were intravaginally infected with *C. muridarum*, the urogenital tract tissues were harvested for observing hydrosalpinx. As shown in Fig. 1, the 11 strains were classified into 3 groups based on their hydrosalpinx severity scores: Mouse strains with severity score of <1 were designated as resistant to hydrosalpinx induction. These mice displayed a hydrosalpinx incidence of 40% or less, including strains NOD/ShiLtJ, DBA/1J, DBA/2J and A/J; mouse strains with hydrosalpinx severity score of  =  or >5 were designated as highly susceptible. Both CBA/J and SJL/J fell into this group and they displayed a hydrosalpinx incidence of 80% or higher; the remaining 5 strains (Balb/c, C57BL/6J, C57BL/10J, C3H/HeJ & C3H/HeN) all developed significant hydrosalpinx with severity scores between 1 and 5 and an incidence of 40% or higher. These 5 strains were designated as susceptible strains. The severity scores from either the susceptible or highly susceptible groups were always significantly higher than those from the resistant group. The gross pathology observation was further supported by microscopic examination of the same mouse oviduct tissues (Fig. 2), which showed significant differences in oviduct lumen dilation between mouse strains in the resistant and those from highly susceptible as well as 3 of 5 mouse strains in the susceptible groups. We further analyzed the relationships between the 4 parameters used for describing mouse oviduct pathology, including hydrosalpinx incidence and severity observed under naked eye and lumenal dilation and inflammation scores evaluated under microscopy (Table 1). We found that these 4 parameters were highly correlative to each other with a Spearman's correlation of >0.8 and p<0.01 (two-tailed Student t test) between any pairs, suggesting that categorization of the 11 strains of mice based on the 4 parameters is appropriate. Since only a subpopulation of women with *C. trachomatis* infection develop tubal factor infertility [11], [12], the distinct phenotypes exhibited by the 11 strains of mice may reflect the different clinical outcomes of women with *C. trachomatis* infection. The question is why these 11 strains displayed different levels of hydrosalpinx in response to the same *C. muridarum* intravaginal infection.

**Table 1:** Correlations between oviduct pathology parameters.

		Gross (naked eye)		Microscopy	
		Incidence	Severity	Lumen dilation	Inflam. score
**Gross (naked eye)**	**Hydrosalpinx incidence**	1			
	**Hydrosalpinx severity**	0.9199* (p<0.01)^#^	1		
**Micro-scopy**	**Lumen dilation**	0.9452 (p<0.01)	0.9066 (p<0.01)	1	
	**Inflammation score**	0.8284 (p<0.01)	0.8744 (p<0.01)	0.9248 (p<0.01)	1

The hydrosalpinx incidence rates and severity scores observed under naked eye (gross pathology from figure 1 above) and oviduct lumen dilation and inflammation scores assessed under microscopy (from figure 2 above) from the 11 strains of mice were analyzed using Spearman's correlation. The results were expressed in Spearman's correlation (*, top) and P value (#, bottom in parentheses) calculated using two-tailed Student t test. Note that these 4 oviduct pathology parameters are highly correlative with each other.

*Spearman's correlation; #p value (Two-tailed Student t test).

### 2. Mouse susceptibility to hydrosalpinx induction is independent of live organism recovery from the lower genital tract or mouse H-2 haplotypes

**Figure 3:**
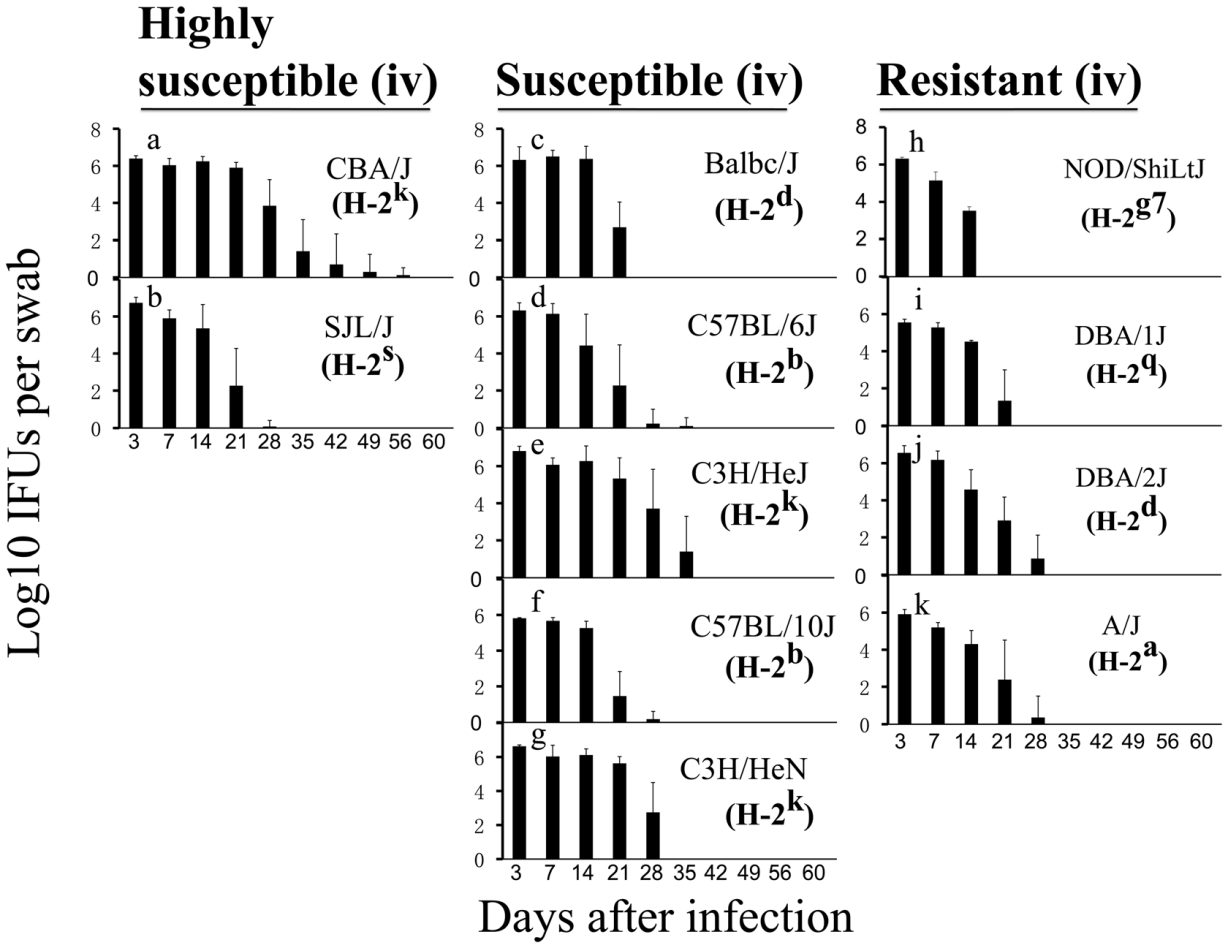
Live *C. muridarum* organism recovery from vaginal swabs along the infection time course. After the intravaginal infection (iv), the same eleven strains of mice described in Fig.1 legend were monitored for live chlamydial organism shedding from the lower genital tract. The number of live organisms recovered from the vagina/cervix swabs is expressed as log_10_ IFUs per swab and displayed along the Y-axis. The eleven strains of mice were classified into 3 distinct groups (panels a and b for highly susceptible, c–g for susceptible and h–k for resistant) as described in Fig. 1 legend. The H-2 haplotype of each mouse strain is also listed in the corresponding bar graph. Note that the susceptibility to hydrosalpinx induction did not correlate with either the level or duration of live organism shedding or the H-2 haplotypes.

To search for factors or correlates associated with the mouse susceptibility to hydrosalpinx development, we compared both live chlamydial organism shedding time courses and mouse H-2 haplotypes among the 11 strains of mice (Fig. 3). The amounts of live organism shedding were similar within the first week after infection between different mouse strains, suggesting that all mice were equally infected. However, starting day 14 post infection, variations in the level of live organism shedding became obvious, leading to varied durations of infection courses in different strains of mice. Some strains cleared or almost cleared infection on days 21 (NOD/ShiLtJ) or 28 (DBA/1J, A/J, Balb/c, C57BL/6J, C57BL/10J and SJL/J) while the others exhibited longer infection time courses up to days 35 (DBA/2J and C3H/HeJ), 42 (C3H/HeJ) or even 56 (CBA/J). However, neither the shedding levels nor the shedding time courses correlated with mouse susceptibility to hydrosalpinx. For example, SJL/J mice were highly susceptible to hydrosalpinx induction but cleared infection as early as day 28 while DBA/2J mice that were resistant to hydrosalpinx induction only cleared infection on day 35. We found no significant correlation of hydrosalpinx severity with the lower genital tract infection status after intravaginal inoculation (Table 2). The lack of correlation was observed both between the 11 different strains and within a given strain of mice. The analysis results were consistent whether the lower genital tract infection was expressed as IFUs at a given time point after infection when the vaginal sample was collected or days required for clearing infection or filled areas under the corresponding live organism shedding curves (Table 2). These correlation analyses have demonstrated that the lower genital tract infection status regardless of how it is expressed cannot predict mouse susceptibility to hydrosalpinx induction by chlamydial infection in the lower genital tract.

**Table 2:** Correlations between hydrosalpinx scores and lower genital tract shedding following *C. muridarum* infection.

		IFUs recovered on						Days to clear	Filled area
		Day 3	Day 7	Day 14	Day 21	Day 28	Day 35		
**Intravaginal**	**11 strains**	0.4292* (p = 0.19)^#^	0.3736 (p = 0.26)	0.6059 (p = 0.048)	0.2096 (p = 0.54)	0.0506 (p = 0.88)	0.4693 (p = 0.15)	0.3678 (p = 0.27)	0.4361 (p = 0.18)
	**C3H/HeJ**	−0.0493 (p = 0.81)	−0.3610 (p = 0.06)	0.2618 (p = 0.19)	0.0654 (p = 0.75)	0.0927 (p = 0.65)	0.0999 (p = 0.62)	na	na
	**C57BL/6J**	0.0853 (p = 0.69)	−0.0217 (p = 0.92)	0.1256 (p = 0.57)	0.0107 (p = 0.96)	−0.2284 (p = 0.29)	0 (p = 1)	na	na
**Intrauterine**	**11 strains**	0.1254 (p = 0.71)	0.0845 (p = 0.81)	0.5 (p = 0.12)	0.1484 (p = 0.66)	0.0616 (p = 0.86)	0.2547 (p = 0.45)	0.5363 (p = 0.09)	0.1412 (p = 0.68)

**Figure 4:**
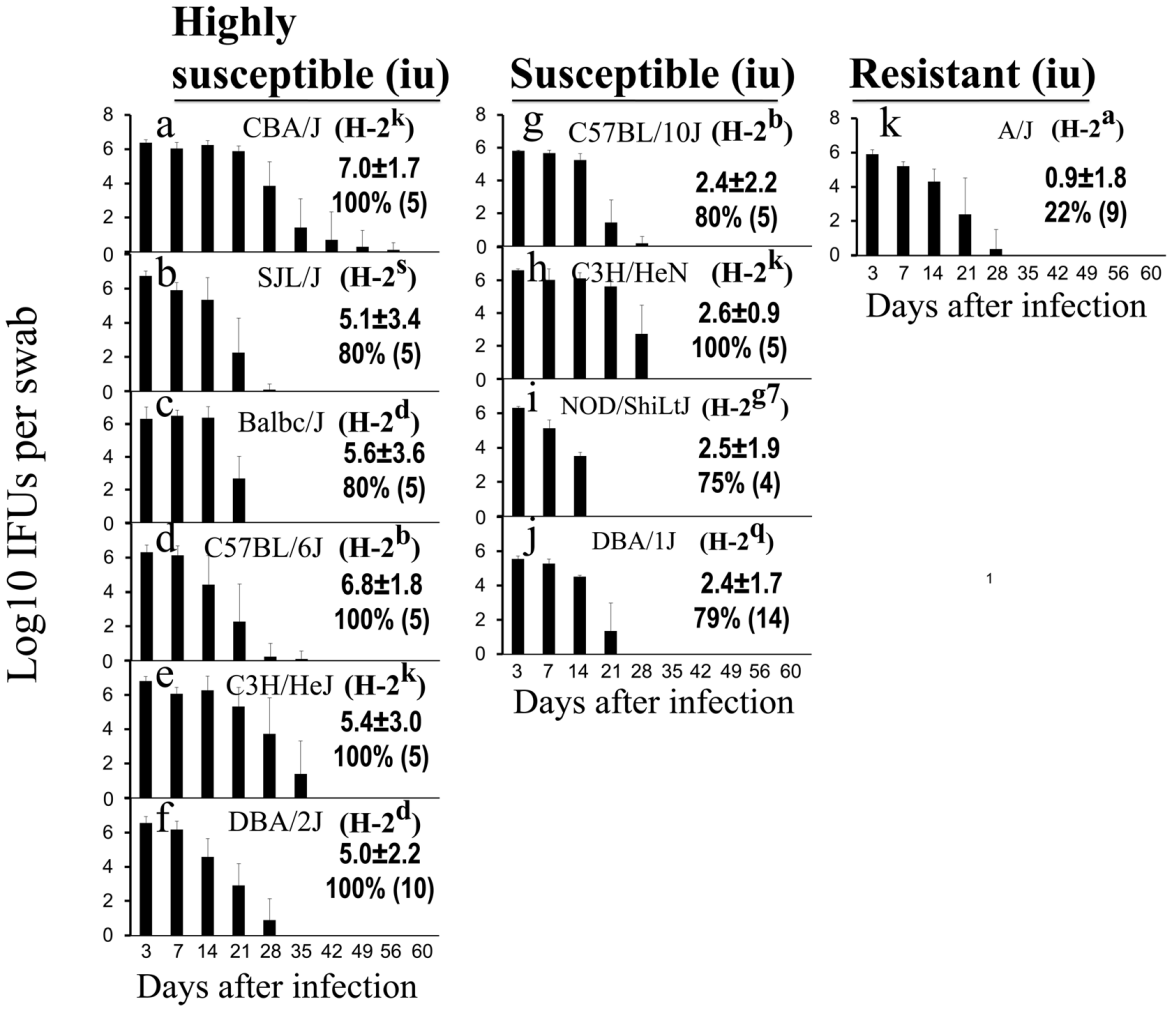
Hydrosalpinx development and live organism shedding following intrauterine infection with *C. muridarum*. Each of the 11 strains of mice was inoculated with 2×10^5^ IFUs of *C. muridarum* via an intrauterine route (iu). Sixty days after inoculation, all mice were sacrificed for observing hydrosalpinx as described in Fig. 1 legend. Based on hydrosalpinx severity scores, the 11 strains were re-categorized into highly susceptible (with a median severity score of 5 or above, panels a-f for mouse strains CBA/J, SJL/J, Balb/cJ, C57BL/6J, C3H/HeJ & DBA/2J respectively), susceptible (<5 and >1, panels g-j for C57BL/10J, C3H/HeN, NOD/ShiLtJ & DBA/1J) or resistant (<1, panel k for A/J). The hydrosalpinx incidences (%) along with total number of mice per strain (in bracket) are listed under the corresponding hydrosalpinx scores. The live organism shedding was monitored post infection as described in Fig. 3 legend and expressed as Log10 IFUs (shown along the Y-axis of each panel). The H-2 haplotypes are listed after the corresponding mouse strain name. Note that the hydrosalpinx severity did not have any obvious correlations with the live organism shedding course or H-2 haplotypes except for H-2^d^. Both Balb/cJ (H-2^d^) and DBA/2J (H-2^d^) were highly susceptible to hydrosalpinx induction.

The hydrosalpinx scores from mice infected with *C. muridarum* via either intravaginal (from figure 1 above) or intrauterine (from figure 4) infections as indicated in the left column was analyzed against the corresponding infection status as shown in the top rows. The infection status was expressed as inclusion-forming units (IFUs) on different days after infection or days required for clearing infection (Days to clear) or areas under the corresponding live organism shedding curves (Filled area, last column). The correlation was analyzed either between different strains (11 strains) or within a given strain (C3H/HeJ or C57BL/6J) of mice as indicated in the 2^nd^ column from the left and the results were expressed as Spearman's correlation (*, top) and P value (#, bottom in parentheses) calculated using two-tailed Student t test. Note that there was no significant correlation between the hydrosalpinx scores and infection status regardless of the types of analyses. na = not analyzed.

*Spearman's correlation; #p value (Two-tailed Student t test); na = not analyzed.

The mouse H-2 haplotypes did not seem to correlate with mouse susceptibility to hydrosalpinx either. Both Balb/cJ and DBA/2J have the H-2^d^ haplotype but the former is significantly more susceptible to hydrosalpinx induction than the latter (76%, 4.8±3.3 versus 20%, 0.6±1.4, p<0.01 for both Fisher's Exact and Wilcoxon). In addition, CBA/J (H-2^k^) and A/J (H-2^a^) both express the H-2^k^ allele at multiple H-2 loci. However, CBA/J mice were highly susceptible while A/J mice were highly resistant to hydrosalpinx induction by *C. muridarum* intravaginal infection.

### 3. Infection at the upper genital tract via intrauterine inoculation enhances hydrosalpinx

Transcervical or intrauterine inoculation can bypass the cervical barrier to allow the inoculated bacteria to direct access to upper genital tract. We delivered *C. muridarum* organisms into the upper genital tract of the 11 strains of mice via intrauterine inoculation and monitored the development of hydrosalpinx (Fig. 4). As expected, most mice developed more significant hydrosalpinx with both increased incidence and severity scores. For example, Balb/cJ, C57BL/6J, C3H/HeJ and DBA/2J all developed hydrosalpinx with a severity score of >5 and were thus re-categorized as highly susceptible strains. The intrauterine inoculation also converted NOD/ShiLtJ and DBA/1J mice from resistant to susceptible phenotype. The vaginal shedding of live organisms after intrauterine infection was monitored, and there was no obvious correlation between vaginal live organism shedding and incidence or severity of hydrosalpinx. For example, DBA/2J and A/J shared a similar shedding course. However, only DBA/2J became highly susceptible while A/J remained resistant to hydrosalpinx induction after intrauterine infection. The lack of correlation between oviduct pathology and live chlamydial organism shedding in the lower genital tract after intrauterine inoculation was confirmed when the relationship of hydrosalpinx severity with live organism shedding was comprehensively analyzed (Table 2). Thus, live organism shedding from the lower genital tract after intrauterine inoculation cannot be used to predict the severity of upper genital tract pathology. Also, mouse strain susceptibility to chlamydial induction of hydrosalpinx via intrauterine infection did not seem to correlate with mouse H-2 haplotypes. For example, both C57BL/6J and C57BL/10J shared the H-2^b^ haplotype but only the C57BL/6J mice were enhanced to the highly susceptible category while the C57BL/10J mice remained susceptible. The same is true with the two H-2^k^ strains C3H/HeJ and C3H/HeN. C3H/HeJ mice were elevated to the highly susceptible group by intrauterine inoculation while C3H/HeN stayed in the susceptible category. Nevertheless, the H-2^d^ haplotype seems to consistently associate with the highly susceptible phenotype with intrauterine infection since Balb/cJ (H-2^d^) and DBA/2J (H-2^d^) both became highly susceptible to hydrosalpinx induction by intrauterine inoculation. In contrast, Balb/cJ mice were susceptible while DBA/2J mice were resistant to hydrosalpinx induction by intravaginal infection (see Fig. 1). These observations suggest that the strain-specific background genes may determine the ascending infection efficiency. Only after the difference in controlling ascending infection was eliminated by intrauterine infection, did the association of the H-2^d^ haplotype with susceptibility to hydrosalpinx induction become obvious.

### 4. Comparison between lower and upper genital tract infections reveals both ascension-dependent and -independent mechanisms for regulating hydrosalpinx

As shown in Table 3, comparing the degree of hydrosalpinx induced via lower versus upper genital tract infections, most mice in the latter category developed more severe hydrosalpinx with higher incidence. Following intravaginal infection, 2 of the 11 strains were highly susceptible, 5 were susceptible and 4 were resistant. However, after intrauterine infection, 6 were highly susceptible and 4 were susceptible while only 1 remained resistant. We found that overall, the hydrosalpinx severity induced by intravaginal infection was highly correlative with that enhanced by intrauterine infection with a Spearman's correlation of 0.7489 and p = 0.008 (two-tailed Student t test, data not shown). This analysis suggested that ascending infection played a significant role in mouse susceptibility to hydrosalpinx development. Nevertheless, only two strains (DBA/1J and DBA/2J) displayed statistically significant differences in hydrosalpinx incidence and severity between intravaginal and intrauterine inoculation. More importantly, the intrauterine inoculation failed to significantly enhance hydrosalpinges in C57BL/10, C3H/HeN (both maintained susceptible phenotype after intravaginal or intrauterine infection) and A/J (maintained a resistant phenotype even after intrauterine infection). These observations suggest that achieving ascending infection by directly inoculating chlamydial organisms into the upper genital tract may not always be sufficient for enhancing pathology in the upper genital tract. These mouse strains may have evolved ascension-independent mechanisms for regulating upper genital tract pathology. Since host inflammatory response is another significant contributor to hydrosalpinx, we compared the cytokine profiles and levels in oviduct between the highly susceptible CBA/1J and the resistant A/J mice (Table 4). We found that 27 of the 32 cytokines were significantly higher in oviducts of CBA/J than those of A/J mice, 14 of which displayed more than 4 fold differences, including chemokines KC (or CXCL1), MIP-2 (Macrophage inflammatory protein 2 or CXCL2), MIG (Monokine induced by gamma interferon or CXCL9), proinflammatory cytokines IL-1α, IL-1β, IL-6, IL-12p70, IL-17, IL-18, T cell cytokines IL-2 & 3 and LIF (leukemia inhibitory factor) and growth factors G-CSF (granulocyte colony-stimulating factor) & VEGF (vascular endothelial growth factor). These chemokines/cytokines/growth factors may contribute to hydrosalpinx development in CBA/J mice.

**Table 3:** Hydrosalpinx induced by lower versus upper genital tract infections with *C. muridarum*.

Mouse strains	Lower genital tract infection (intravaginal inoculation)			Upper genital tract infection (intrauterine inoculation)		
	^Incidence (n)^	^Severity^	^Susceptibility^	^Incidence (n)^	^Severity^	^Susceptibility^
**^CBA/J (H-2k)^**	87% (15)	5.1±2.8	H (Score: = or >5)	100%(5)	7.0±1.7	^H^
**^SJL/J (H-2s)^**	80% (15)	5.3±3.2		80%(5)	5.1±3.4	
**^Balb/cJ (H-2d)^**	76% (17)	4.8±3.3	S (Score: >1 to <5)	80% (5)	5.6±3.6	
**^C57BL/6J (H-2b)^**	78% (23)	4.1±2.9		100% (5)	6.8±1.8	
**^C3H/HeJ (H-2k)^**	80% (10)	4.0±2.7		100% (5)	5.4±3.0	
**^C57BL/10J (H-2b)^**	60% (5)	2.4±3.2		80% (5)	2.4±2.2	^S^
**^C3H/HeN (H-2k)^**	40% (5)	2.0±2.4		100% (5)	2.6±0.9	
**^NOD/ShiLtJ (H-2g7)^**	40% (5)	0.8±1.0	R (Score: <1)	75% (4)	2.5±1.9	
**^DBA/1J (H-2q)^**	29% (17)	0.4±0.6		79% (14)*	2.4±1.7**	
**^DBA/2J (H-2d)^**	20% (15)	0.6±1.4		100% (10)*	5.0±2.2**	^H^
**^A/J (H-2a)^**	10%(10)	0.4±1.3		22% (9)	0.9±1.8	^R^

The 11 strains of mice listed along with their H-2 haplotypes in the left column were categorized into 3 groups under lower and upper genital infection respectively. Mice with hydrosalpinx severity scores of 5 or >5 were categorized as highly susceptible (H), between 5 and 1 as susceptible (S) and below 1 as resistant (R) to hydrosalpinx induction. Note that introduction of *C. muridarum* organisms directly into upper genital tract via intrauterine inoculation enhanced hydrosalpinx incidence and/or severity in most mice when compared with intravaginal inoculation at the lower genital tract. The mouse strains with hydrosalpinx most significantly enhanced by intrauterine inoculation are DBA/1J and DBA/2J. *p<0.05, **p<0.01.

**Table 4:** Cytokines from oviduct tissue harvested 14 days after intravaginal infection.

Strain	CBA/J (n = 5)	A/J (n = 5)	Ratio (CBA/J v.s. A/J)	P value ^(Score: <1)^ (t-Test)
**IL-1α**	5822.3±660.3	1155.5±1367.1	**5.0**	0.00
**IL-1β**	7813.6±2270.9	1541.1±1896.4	**5.1**	0.00
**IL-2**	20.6±2.2	5.0±11.0	**4.1**	0.01
**IL-3**	14.7±3.2	3.1±3.1	**4.7**	0.00
**IL-4**	44.6±6.3	15.3±15.9	2.9	0.01
**IL-5**	35.2±5.3	15.8±9.5	2.2	0.00
**IL-6**	302.1±82.5	23.7±31.8	**12.8**	0.00
**IL-9**	494.5±42.2	451.7±53.8	1.1	
**IL-10**	75.5±12.2	25.0±17.7	3.0	0.00
**IL-12 (p40)**	510.2±122.7	227.9±100.9	2.2	0.00
**IL-12 (p70)**	389.4±36.4	81.0±92.3	**4.8**	0.00
**IL-13**	643.3±105.2	448.6±166.5	1.4	
**IL-17**	357.1±113.1	35.6±22.6	**10.0**	0.00
**Eotaxin**	1204.4±311.1	662.8±351.7	1.8	0.04
**G-CSF**	9072.9±5983.9	1216.2±2080.6	**7.5**	0.02
**GM-CSF**	173.4±21.4	98.4±61.3	1.4	0.02
**IFNγ**	228.7±43.0	110.6±73.0	2.1	0.01
**MCP-1**	6309.6±1584.0	1633.2±1026.9	3.9	0.00
**MIP-1α**	575.9±187.0	274.6±234.5	2.1	
**MIP-1β**	310.4±64.2	125.4±59.2	2.5	0.00
**RANTES**	824.0±93.8	516.9±362.7	1.6	
**TNFα**	139.5±30.0	58.5±45.4	2.4	0.01
**IL-15**	601.6±146.9	245.6±168.8	2.4	0.01
**KC**	1176.9±415.2	159.3±156.6	**5.9**	0.00
**IL-18**	640.9±254.9	133.8±83.2	**4.8**	0.00
**FGF-basic**	2703.0±300.5	1288.8±290.3	2.1	0.00
**LIF**	202.2±66.2	44.0±37.4	**4.6**	0.00
**M-CSF**	369.3±62.9	140.6±106.1	2.6	0.00
**MIG**	197599±61399.3	18387.7±21120.6	**10.7**	0.00
**MIP-2**	5840.9±4125.5	1296.2±1324.1	**4.5**	0.04
**PDGF-BB**	0	0		
**VEGF**	3022.4±1099.4	596.1±509.1	**5.1**	0.00

Oviduct tissue homogenates were made from highly susceptible strain CBA/J (n = 5) and resistant strain A/J (n = 5) on day 14 after intravaginal inoculation for simultaneous measurement of 32 cytokines using a multiplex bead array assay. All cytokines were expressed in pg/ml as mean plus/minus standard deviation. The means from the two mouse strains were used for calculating ratio and Student t-Test. A ratio of CBA/J versus A/J above 4 is highlighted in bold face. Note that 27 of the 32 cytokines were significantly higher in oviducts of highly susceptible CBA/J than those of resistant A/J mice, 14 of which displayed more than 4 folds difference.

## Discussion

Preventing upper genital tract pathology caused by lower genital tract infections is essential for maintaining normal function of the female reproductive system. The upper genital tract pathology hydrosalpinx is a hallmark of tubal factor infertility, which can be induced via lower genital tract infection with *C. trachomatis* in women and *C. muridarum* in female mice. To understand the mechanisms of hydrosalpinx and to reveal host strategies for preventing hydrosalpinx, we compared 11 strains of mice for hydrosalpinx induction by *C. muridarum* infection. Three significant findings have been made in the current study. First, following an intravaginal infection, the 11 strains of mice fell into 3 categories: highly susceptible, susceptible and resistant to hydrosalpinx induction. The diverse mouse susceptibility to hydrosalpinx induction by intravaginal infection with *C. muridarum* may reflect the different clinical outcomes of *C. trachomatis*-infected women. Understanding the mechanisms involved in hydrosalpinx development in each category of mouse strains may allow us to acquire knowledge relevant for understanding how different women develop different outcomes upon *C. trachomatis* infection. Second, to further differentiate the roles of the interactions between chlamydial ascending infection and host control of ascension from those independent of ascension in hydrosalpinx development, we directly inoculated *C. muridarum* organisms to the upper genital tract of the 11 strains of mice via intrauterine injection to bypass the requirement for ascending infection. Most mice developed more severe hydrosalpinx following upper genital tract infection, suggesting that ascending infection is a key step in determining host susceptibility to hydrosalpinx development. Finally, three mouse strains (C57BL/10J, C3H/HeN and A/J) only experienced minimal hydrosalpinx enhancement despite the upper genital tract inoculation, indicating that ascension-independent mechanisms may play a more important role in regulating hydrosalpinx in these mice. Thus, different strains of mice may have not only developed different susceptibility to hydrosalpinx induction but also evolved different mechanisms for regulating upper genital tract pathology. Although ascending infection is considered necessary for hydrosalpinx induction, it alone is not sufficient.

It is estimated that 10 to 15% women with *C. trachomatis* infection in the lower genital tract (if untreated) may develop upper genital tract pathology (http://www.cdc.gov/std/infertility; ref:[11], [12]. Similarly, different inbred strains of mice displayed distinct susceptibilities to hydrosalpinx induction by *C. muridarum* infection in the lower genital tract. Among the 11 strains screened, only CBA/J and SJL/J mice (representing ∼18% of the 11 strains) were highly susceptible to hydrosalpinx induction by the lower genital tract inoculation with *C. muridarum*, which may be equivalent to the *C. trachomatis*-infected women who develop upper genital pathology. Why are these two strains highly susceptible to hydrosalpinx induction? The susceptibility does not seem to correlate with the live organism shedding courses. The overall levels of shedding were similar among all 11 strains of mice. Although the shedding time from CBA/J was longer, the SJL/J shedding time course was very short, shorter than those of mice with a resistant phenotype. More importantly, there was no overall correlation between hydrosalpinx severity and live organism shedding from the lower genital tract. Thus, we can conclude that mouse susceptibility to hydrosalpinx induction is not in proportion with the mouse susceptibility to chlamydial lower genital tract infection. Both CBA/J and SJL/J may lack the ability to effectively control ascending infection, which may be a key factor for explaining their high susceptibility to hydrosalpinx development following a lower genital tract infection. This assumption is supported by the observation that both strains developed maximal levels of hydrosalpinx when the initial infection was introduced to either lower or upper genital tracts. We can also assume that both strains might have developed maximal levels of hydrosalpinx-causing inflammation regardless of the infection routes. However, neither the inflammation hyper-responsiveness nor the hydrosalpinx susceptibility is linked to H-2 haplotypes since CBA/J is H-2^k^ while SJL/J is H-2^s^ and these two haplotypes share no similar loci. Further characterization of oviduct infection and inflammation in these two strains may shed new light on the molecular events responsible for the high susceptibility to hydrosalpinx induction following a lower genital tract infection.

Most *C. trachomatis*-infected women do not develop upper genital tract pathology. Obviously, many factors can contribute to the “resistant” phenotype. What we want to emphasize here are the biologically relevant mechanisms. We hypothesize that different women may use different mechanisms for preventing upper genital tract pathology, which is supported by the findings in the current study that different strains of mice may use different mechanisms, including ascension-dependent and -independent mechanisms, to prevent pathology development in the upper genital tract. First, among the 5 susceptible strains defined under intravaginal inoculation, 3 (Balb/cJ, C57BL/6J & C3H/HeJ) were elevated to highly susceptible by intrauterine inoculation while 2 (C57BL/10J & C3H/HeN) remained susceptible. These observations suggest that Balb/cJ, C57BL/6J & C3H/HeJ mice are more dependent on the control of ascending infection for preventing upper genital tract pathology while C57BL/10J and C3H/HeN may have evolved ascension-independent mechanisms for regulating upper genital tract pathology. It is not clear whether C57BL/10J and C3H/HeN mice are less able to develop hydrosalpinx-causing inflammation or more capable of suppressing hydrosalpinx-causing inflammation in oviducts. Second, among the 4 resistant strains defined under intravaginal inoculation, 3 were elevated to either susceptible (NOD/ShiLtJ & DBA/1J) or highly susceptible (DBA/2J) by intrauterine inoculation while A/J mice remained resistant. These observations suggest that NOD/ShiLtJ, & DBA/1J, and especially DBA/2J, are highly dependent on the blockade of ascension for preventing upper genital tract pathology. In contrast, A/J mice may have developed ascension-independent mechanisms for preventing upper genital tract pathology.

The next question is the nature of ascension-dependent and -independent mechanisms. The ascension-dependent mechanism is based upon how mice prevent lower genital tract microbes from ascending to the upper genital tract. The function of cervical barrier is critical for blocking microbial ascension. The cervical barrier may consist of physical barriers such as cervical plug, chemical barriers such as lysozyme, complement and other innate immune effector molecules, and microbiological barriers or the cervical microbiome. Different mouse strains may vary in the composition of these barrier effects. For example, the function of the cervical barrier in CBA/1J and SJL/J mice may be very inefficient in blocking *C. muridarum* ascension while the cervical barrier of DBA/1J and DBA/2J may be very effective in preventing *C. muridarum* ascension. The adaptive immunity induced by the genital tract infection may also contribute to the blockade of *C. muridarum* ascension one week after the initial infection.

The ascension-independent mechanisms may require the host to develop and regulate hydrosalpinx-causing inflammation following chlamydial infection. The intrauterine inoculation has provided us an ideal opportunity to evaluate the role of ascension-independent mechanisms in regulating hydrosalpinx since it allows chlamydial organisms to bypass the requirement for ascending to the oviduct where hydrosalpinx occurs. Both CBA/J and SJL/J mice developed maximal levels of hydrosalpinx following either intravaginal or intrauterine infection, suggesting that these mice are hyper-responsive to chlamydial stimulation in developing hydrosalpinx and/or have little ability to negatively regulate oviduct inflammation. The conversion of DBA/2J mice from resistant phenotype after intravaginal infection to highly susceptible phenotype after intrauterine infection is another example that DBA/2J mice posses the ability to develop hydrosalpinx with little to no ability to down regulate hydrosalpinx-causing inflammation in oviduct. On the contrary, C57BL/10, C3H/HeN and A/J mice maintained the same levels of susceptibility to hydrosalpinx induction by either intravaginal or intrauterine infection, suggesting that these mice either fail to develop hydrosalpinx-causing inflammation or negatively regulate hydrosalpinx-causing inflammation in the oviduct. These hypotheses are supported by the finding that CBA/J mice produced significantly more cytokines than A/J mice did (see Table 2). It is worth noting that A/J mice are defective in complement 5 (C5; ref: [18], [19] and C5 has been known to play an important role in inflammatory pathology [20], [21]. It will be interesting to test whether C5 deficiency is responsible for the reduced inflammatory cytokine responses and lack of hydrosalpinx in A/J mice after chlamydial infection. However, the resistant DBA/2J mice are also defective in C5 [22] and DBA/2J mice were induced to develop severe hydrosalpinx by intrauterine inoculation with *C. muridarum*, suggesting that hydrosalpinx can be induced in the absence of C5 in the context of DBA/2J background. Obviously, the role of C5 in hydrosalpinx development needs to be addressed by comparing wild type and C5 deficient mice in the same background. Regardless whether C5 is required for chlamydial induction of hydrosalpinx, the fact that the oviduct cytokines in A/J mice were significantly lower than those of CBA/J after chlamydial infection suggests that A/J mice are either unable to develop a robust hydrosalpinx-causing inflammation or possess mechanisms for suppressing hydrosalpinx-causing inflammation. Therefore, A/J mice can serve as a relevant model for further understanding ascension-independent mechanisms involved in hydrosalpinx development.
